# A Prediction Framework of Apple Orchard Yield with Multispectral Remote Sensing and Ground Features

**DOI:** 10.3390/plants15020213

**Published:** 2026-01-09

**Authors:** Shuyan Pan, Liqun Liu

**Affiliations:** College of Information Science and Technology, Gansu Agricultural University, Lanzhou 730070, China

**Keywords:** apple orchard, remote sensing, YOLO, yield prediction, proportional correction

## Abstract

Aiming at the problem that the current traditional apple yield estimation methods rely on manual investigation and do not make full use of multi-source information, this paper proposes an apple orchard yield prediction framework combining multispectral remote sensing features and ground features. The framework is oriented to the demand of yield prediction at different scales. It can not only realize the prediction of apple yield at the district and county scales, but also modify the prediction results of small-scale orchards based on the acquisition of orchard features. The framework consists of three parts, namely, apple orchard planting area extraction, district and county large-scale yield prediction and small-scale orchard yield prediction correction. (1) During apple orchard planting area extraction, the samples of some apple planting areas in the study area were obtained through field investigation, and the orchard and non-orchard areas were classified and discriminated, providing a spatial basis for the collection of subsequent yield prediction-related data. (2) In the large-scale yield prediction of districts and counties, based on the obtained orchard-planting areas, the corresponding multispectral remote sensing features and environmental features were obtained using Google Earth engine platform. In order to avoid the noise interference caused by local pixel differences, the obtained data were median synthesized, and the feature set was constructed by combining the yield and other information. On this basis, the feature set was divided and sent to Apple Orchard Yield Prediction Network (APYieldNet) for training and testing, and the district and county large-scale yield prediction model was obtained. (3) During the part of small-scale orchard yield prediction correction, the optimal model for large-scale yield prediction at the district and county levels is utilized to forecast the yield of the entire planting area and the internal local sampling areas of the small-scale orchard. Within the local sampling areas, the number of fruits is identified through the YOLO-A model, and the actual yield is estimated based on the empirical single fruit weight as a ground feature, which is used to calculate the correction factor. Finally, the proportional correction method is employed to correct the error in the prediction results of the entire small-scale orchard area, thus obtaining a more accurate yield prediction for the small-scale orchard. The experiment showed that (1) the yield prediction model APYieldNet (MAE = 152.68 kg/mu, RMSE = 203.92 kg/mu) proposed in this paper achieved better results than other methods; (2) the proposed YOLO-A model achieves superior detection performance for apple fruits and flowers in complex orchard environments compared to existing methods; (3) in this paper, through the method of proportional correction, the prediction results of APYieldNet for small-scale orchard are closer to the real yield.

## 1. Introduction

Multispectral satellite remote sensing image is a kind of remote sensing data that uses the sensors carried on the satellite to obtain the land surface information in multiple specific bands at the same time, including visible light, near-infrared, and short infrared bands. This image can not only record the color information visible to the human eye, but can also capture the characteristics of invisible ground features such as plant health, water distribution, soil moisture, and so on. Multiband satellite remote sensing data contain a large amount of spectral information, which can be used to calculate different vegetation indexes (VI) for different agricultural research. Using multispectral satellite remote sensing images to predict orchard yield can achieve good results in a large range of districts and counties, but it will produce large errors if it is used in a relatively fine and small range of yield prediction. Therefore, when using multispectral satellite remote sensing images for small-scale yield prediction, it is necessary to introduce the corresponding ground features to supplement, so as to further modify the prediction results.

In response to the current problem of relying on manual surveys for apple yield prediction, which is time-consuming and laborious, this paper proposes an apple orchard yield prediction framework that combines multispectral remote sensing features and ground features. This framework consists of three parts, namely apple orchard planting area extraction, large-scale yield prediction for districts and counties, and small-scale orchard yield prediction correction.

### 1.1. Related Work

Orchard yield prediction techniques can generally be divided into two main categories: direct and indirect approaches.

(1) Direct prediction

Most direct prediction methods rely on object detection in the field of computer vision to conduct on-site counting of orchards, establish a connection between the detection results and the final yield, and thus achieve yield prediction. Häni, Nicolai et al. proposed a modular end-to-end system for estimating apple orchard yield, which first determines the optimal fruit detection and counting method, and then estimates yield based on the number of fruits. The results show that this method can achieve high yield estimation accuracy [1]. Jamil Ahmad et al. used drone imaging for automated yield estimation in orchards. After obtaining the number of fruits and their health level through the detection network, factors such as canopy area are added to establish a linear regression model to learn the relationship between different inputs and the predicted yield, ultimately achieving yield prediction [2]. Gao et al. proposed a fruit counting and yield estimation method based on orchard videos. The number of fruits detected by this method can further provide data for yield prediction tasks [3]. Sun et al. proposed a lightweight apple detection method called YOLOv5-PRE, and the results showed that the method is suitable for porting to embedded devices and has strong robustness to lighting conditions, providing effective methods and data support for yield prediction [4]. Mirhaji, Hamzeh et al. designed a yield estimation method using transfer learning to estimate orange yield, and used a trained deep learning model to detect and count orange tree images [5]. Wang et al. proposed a lightweight model for detecting dense plums in orchards to address the problem of poor detection results caused by small plum fruits. The results showed that the model has strong robustness to factors such as lighting in actual environments and can provide reference data for subsequent yield estimation [6]. Luis Gonzalez Nieto et al. evaluated and compared the accuracy of different commercial computer vision systems in yield estimation in 23 orchards [7]. Liu et al. designed a detection model to address the problem of poor detection performance caused by the small size and difficulty in distinguishing color from the background of green crisp plum fruits. The results showed that the model could obtain better recognition results and provide data support for subsequent yield estimation [8]. Marcelo H. Amaral et al. used calipers to measure the length, width, and thickness of labeled mango fruits on trees at different stages, and based on this, established a linear regression model to predict the final yield [9]. Manal El Akrouchi et al. proposed a green citrus fruit detection framework and designed a dual image training strategy. Close up images and whole tree images were used to train the detection model and conduct practical application testing, respectively. To improve the accuracy of whole tree detection, a slicing method was used to divide high-resolution images into small blocks to capture detailed information. The results showed that slicing technology can improve model performance and provide technical support for yield prediction [10]. Xiong et al. proposed a novel method combining unmanned aerial vehicle remote sensing and deep learning for single tree detection and spatial distribution mapping. This method can count the number and planting area of fruit trees within a specified range, providing key data for intelligent orchard management such as yield prediction [11].

(2) Indirect prediction

Most indirect prediction methods are based on spectral information, supplemented by orchard morphological features such as canopy area, for yield prediction. Multispectral images are obtained through satellite remote sensing or unmanned aerial vehicle remote sensing and calculating vegetation indices, while climate and other features are added to form multi-source feature inputs, and establishing a connection with the final yield to achieve yield prediction. Abdellatif Moussaid et al. combined spectral information from satellite imagery and historical field data to train a machine learning model for predicting citrus fruit yield. Experimental results showed that this method can achieve better prediction results and can be applied to other tree crops for yield prediction [12]. Zhang et al. used climate factors and remote sensing vegetation index features to train stochastic gradient boosting and proposed a new method for predicting almond yield. At the same time, the experiment analyzed several key factors affecting almond orchard yield [13]. Kim, Jaehong et al. proposed a new method for predicting citrus orchard yield based on unmanned aerial vehicles and hyperspectral technology. A linear regression function was established between the extracted citrus pixel values and the actual harvest weight. Thirty citrus trees were randomly selected in three experimental areas for verification, and the results showed that the linear regression function could obtain good fitting results [14]. Benjamin Adjah Torgbor et al. used six machine learning methods to predict mango yield based on multi-vegetation indices and climate variables, and analyzed the key factors affecting yield. The results verified the feasibility of using remote sensing technology to predict farm level or district level yield [15]. Chen et al. established an ensemble learning model based on the spectral and morphological characteristics of apple tree canopy, combined with support vector regression and K-nearest neighbor. The model was used for prediction at orchard scale and individual tree scale, respectively. The results showed that this method can accurately extract the morphological and spectral features of apple trees, and further utilize multi-source remote sensing data to predict yield [16]. Tang et al. designed a convolutional neural network model with spatial attention mechanism based on summer aerial images containing four spectral bands. They used the spectral reflectance information in the images to estimate tree-level almond yield. While achieving good prediction results, they found that the reflectance information in the red edge band was the most important feature in the yield estimation process [17]. Luz Angelica Suarez et al. calculated vegetation index information in the study area based on Landsat satellite data and used it to predict citrus yield. The results showed that this method can obtain relatively accurate predicted yield, providing a new understanding of seasonal changes in citrus yield [18]. Javier E. Gómez-Lagos et al. used normalized vegetation index and artificial neural network to predict fruit yield. The results showed that this spectral-based yield prediction method can advance the prediction time by more than two months and can be used to assist in the coordinated design of various stages of the fruit supply chain [19]. Muhammad Munir Afsar and colleagues designed three specialized vegetation indices for mangoes based on their characteristics after predicting the health of individual mango trees using high-resolution multispectral drone imagery. They also combined data such as tree age, variety, and canopy area to estimate yield through a decision tree algorithm [20]. Muhammad Moshiur Rahman and his team obtained enhanced vegetation indices for avocado orchards through Sentinel-2 products and analyzed their changing trends. The study further demonstrated that this information could be utilized to develop phenology-based yield prediction models [21].

### 1.2. Existing Problems

(1) Traditional apple orchard yield prediction mostly relies on manual counting, which is time-consuming, labor-intensive, difficult, prone to errors, and does not fully utilize multi-source remote sensing and environmental features, resulting in insufficient accuracy of prediction results.

(2) Complex conditions such as overlapping tree canopies and variable illumination frequently occur in orchards. Existing object detection methods still exhibit limitations in accuracy and robustness under such circumstances.

(3) The models trained on data from districts or larger scales often exhibit systematic biases when transferred to local small-scale orchards, thereby affecting the accuracy of yield prediction.

### 1.3. Contributions

The main innovations and contributions of this work are presented as follows:

(1) By comprehensively utilizing spectral information, vegetation indices, elevation data, and texture features, and using the cultivated land areas from land cover type products as the base map, the orchard planting areas are extracted.

(2) Using remote sensing multispectral data and environmental data as inputs, the APYieldNet model was designed for apple yield prediction.

(3) A YOLO-A model is proposed for detecting apple flowers and fruits in complex orchard scenes, providing a more reliable detection foundation for yield estimation.

(4) The optimal APYieldNet model trained on large-scale data from districts and counties will be applied to small-scale orchards, and the prediction results of small-scale orchards will be further corrected using proportional correction based on the ground features of small-scale orchards.

### 1.4. The Structure of the Paper

The paper is structured into six sections. Section 1 introduces the study, reviewing the relevant literature and outlining the contributions. Section 2 details the research area and datasets. Section 3 presents the proposed yield prediction method. Section 4 reports the experimental results using tables and figures, followed by a detailed discussion in Section 5. The Section 5 also summarizes the experimental findings, and Section 6 concludes the paper, highlighting limitations and proposing future research directions.

## 2. Materials

### 2.1. The Remote Sensing Data and Environmental Data

The study area of this paper covers Tianshui City, Qingyang City, and Pingliang City in Gansu Province. Tianshui City falls under the Qinling climate zone, situated at the junction of subtropical and warm temperate zones. The precipitation in the north is significantly less than that in the south, with the annual precipitation mainly concentrated from May to September.

The corresponding data for the three major apple-producing cities in Gansu Province, China (Tianshui City, Pingliang City, and Qingyang City) from April to October from 2019 to 2023, obtained through the Google Earth Engine (GEE) platform, is as follows: Sentinel-2 data, with cloud cover controlled below 20%; 11 types of environmental data from FLDAS. As shown in Table 1. The Sentinel-2 data used in this paper underwent atmospheric correction. After obtaining the Sentinel-2 image data, first, the region of interest was cropped based on the vector boundary of the research area and remove irrelevant area information; secondly, images with overall cloud cover greater than 20% were removed and QA60 quality control bands was used to remove clouds from each image; then, median synthesis was performed on the filtered images on a monthly basis and they were exported; finally, batch mask extraction was performed using ArcGIS (10.8) software and the results summarized into a feature sequence set.

This paper obtained remote sensing data and environmental data corresponding to 22 districts and counties in Gansu Province from 2019 to 2023. After merging Sentinel-2 data, environmental data, and ground statistical data into a feature sequence set, a small amount of Gaussian noise (with the noise amplitude set to 1% of the standard deviation) was introduced based on the standard deviation of different features. Secondly, the constrained Mixup strategy was adopted, where two samples were randomly selected in the same district or county. The mixing coefficients were obtained through sampling from a beta distribution, and a linear combination was performed on the features and yield labels. At the same time, the label difference between the participating samples was limited to no more than 10% of the standard deviation of yield labels within the county to avoid obtaining unreasonable samples. Finally, a tabular variational autoencoder was used to learn the data distribution pattern, and iterative sampling was performed until the required number of new samples conforming to the distribution was generated. These new samples were then merged with the original data to form an expanded and complete dataset. The data from 2023 were used as the test set, and the rest were divided into training and validation sets in an 8:2 ratio.

### 2.2. Apple Fruit and Flower Target Detection Data

This study used the Canon PowerShot SX210 IS telephoto color digital camera (Canon Inc., Tokyo, Japan) to capture RGB images corresponding to the four stages of apple growth (flowering period, young fruit period, fruit swelling period, and ripening period). The core parameters of the camera are shown in Table 2. In the natural environment of the orchard, multi-angle photos were taken with naturally growing apples and apple flowers as the target, and the shooting distance was controlled within 0.5–1.5 m. To unify the experimental input and reduce computational overhead, all image resolutions were uniformly adjusted to 640 × 480 pixels in subsequent processing. The collection location was the orchard of Tianshui Fruit Tree Research Institute in Gansu Province. The data collection times were 10:00, 12:00, 15:00, and 18:00 throughout the day. Images were taken under both forward and reverse lighting conditions. The collected images cover different poses, sizes, lighting conditions, occlusion situations, etc. Due to the main objective of this study being to achieve automatic detection of apple fruits, rather than precise classification based on lighting conditions or fruit development stages, no explicit grouping labeling was performed on the data for lighting or development stages.

For apple flower data, more than 2600 images were collected and X-AnyLabeling (2.5.4) was used to label the fully bloomed flowers. Using the Annotations library, we selected illumination perturbation and geometric transformation methods for data augmentation, resulting in a total of 6826 images. These were divided into a training set (5460 images), validation set (683 images), and test set (683 images) in an 8:1:1 ratio.

By training apples of different colors and developmental stages as a single category, the model can learn more robust fruit appearance and structural features, thereby improving its generalization ability in complex orchard environments. More than 3000 images were collected and labeled using X-AnyLabeling (2.5.4). Simultaneously, 1000 images were selected from each of the datasets AppleBBCH76 and AppleBBCH81 [22] to form the original dataset. Using the Annotations library, we selected illumination perturbation, occlusion simulation, and geometric transformation methods for data augmentation to increase sample diversity and improve the model’s detection robustness under different lighting conditions, occlusion situations, and shooting angles. As a result, we obtained a total of 10,914 images, which were divided into a training set (8732 images), validation set (1091 images), and test set (1091 images) in an 8:1:1 ratio.

### 2.3. Yield Forecast Related Data

Apple yield and planting area data were obtained for different districts and counties from 2019 to 2023 through the Gansu Statistical Yearbook published by the Gansu Provincial Bureau of Statistics.

In order to achieve small-scale orchard yield prediction correction, in the apple maturity stage of 2023, within the planting area of Tianshui Fruit Tree Research Institute, areas with good growth and no obvious pests and diseases were selected as the local sampling areas, and single fruit tree photos were taken of 30 apple trees in the area. The number of fruits was identified and combined with empirical single fruit weight to obtain the unit yield of the local sampling area. For the entire planting area, after fruit harvesting and precise weighing, the unit yield (kg/mu) within the entire planting area of the research institute was obtained by combining the planting area.

## 3. Methods

### 3.1. A Prediction Framework of Apple Orchard Yield with Multispectral Remote Sensing and Ground Features

**Figure 1:**
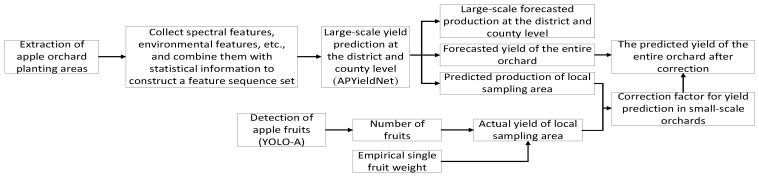
Yield forecasting framework.

The detailed framework for yield prediction in this paper is shown in Figure 1. Firstly, obtain a relatively reliable apple orchard planting area within the study region, and collect corresponding spectral characteristics, environmental characteristics, and statistical information. Secondly, predict the large-scale yield of districts and counties, the yield of the entire orchard of the Tianshui Fruit Research Institute, and the yield of local sampling areas within the orchard through the APYieldNet model. Then, identify the number of fruits on individual fruit trees within the local sampling area through YOLO-A, and obtain the total number of fruits within the local sampling area. Combine the empirical single fruit weight with the area of the sampling area to obtain the actual yield of the local sampling area. Next, calculate the small-scale orchard yield prediction correction factor by comparing the predicted yield of the local sampling area with the actual yield of the local sampling area. Finally, use the above correction factor to correct the predicted yield of the entire orchard to obtain more reliable prediction results.

### 3.2. Mapping of Apple Orchard Cultivation Areas

**Figure 2:**
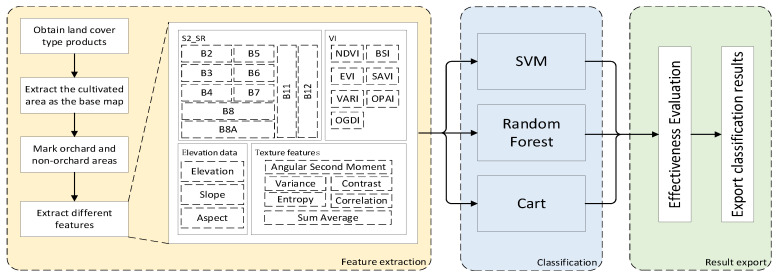
Extraction of apple orchard planting areas.

In this study, the extraction of apple orchard areas is divided into three modules: feature extraction, classification, and result export. In the feature extraction module, according to the literature [23], apple orchards are predominantly located within cultivated agricultural regions in the study area. Therefore, to generate a more precise spatial distribution map of apple orchards within the study area, firstly, the arable land area from the land cover-type product was selected as the base map. Combined with Google Earth imagery, 200 apple planting areas and 200 other areas were labeled from regions such as the Tianshui Fruit Tree Research Institute through visual interpretation. Then, various spectral reflectance information, vegetation indices, texture features, and elevation information of the marked points were obtained as a feature set. The spectral reflectance information includes three visible-light bands (B2, B3, B4), three red-edge bands (B5, B6, B7), two near-infrared bands (B8, B8A), and two short-wave infrared bands (B11, B12). The vegetation indices included Normalized Difference Vegetation Index (NDVI), Enhanced Vegetation Index (EVI), Bare Soil Index (BSI), Soil-Adjusted Vegetation Index (SAVI), Visible Atmospheric Resilience Index (VARI), Orchard Phenological Accumulation Index (OPAI), and Orchard Greenness Difference Index (OGDI). Using the B8 band to construct a gray level co-occurrence matrix, the extracted texture features included Angular Second Moment, Variance, Contrast, Entropy, Correlation, and Sum Average. Elevation information included elevation, slope, and aspect. In the classification module, supervised pixel-level classification was performed using Random Forest, Support Vector Machine, and Classification and Regression Tree, respectively. In the result export module, the differences between the apple-planting area areas obtained by different classification methods and the actual planting area were compared, and the grid map of apple orchard planting areas with the smallest difference was exported. The detailed workflow is illustrated in Figure 2.

### 3.3. Large-Scale Yield Prediction at District and County Levels

**Figure 3:**
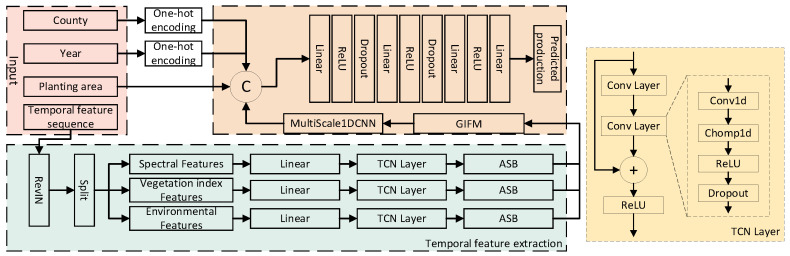
Yield prediction model.

To fully utilize spectral features, vegetation index features, and environmental features to achieve yield prediction tasks for apple orchards, this paper designs a yield prediction model named APYieldNet, as depicted in Figure 3. The input data is divided into three statistical features—county code, year code, and planting area, as well as the temporal features of apple growth period composed of spectral features (B2, B4, B8, B11, B12), 11 environmental data shown in Table 1, and vegetation index features (NDVI, EVI).

For the temporal characteristics of the input, first, normalization is performed using RevIN to adapt to the numerical offset problem caused by non-stationary distributions. Secondly, in response to the heterogeneity in dynamic patterns between different categories of temporal features, each category of temporal feature is independently modeled through TCN layers. On this basis, in order to further enhance the expression ability of key changes in temporal features, ASB [24] is introduced to perform frequency domain transformation on the time series, combined with learnable frequency domain weights and adaptive high-frequency selection mechanism, to strengthen important frequency components and highlight temporal dynamic information closely related to crop growth process. Then, different categories of temporal features are fed into the Grouped Interaction Fusion Module (GIFM) for feature fusion, and multi-scale 1DCNN is used to model the fused features to capture temporal information under different temporal receptive fields. Multi-scale 1DCNN integrates features of different scales through adaptive weighting, thereby enhancing the model’s ability to represent key temporal scale features. Finally, after being concatenated with statistical features, they are sent to the regression head to obtain the final prediction result.

**Figure 4:**
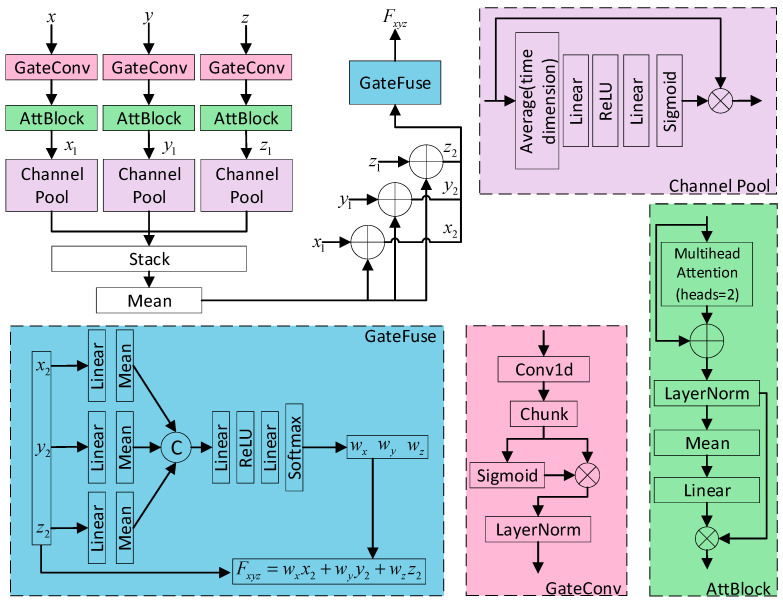
The structure diagram of GIFM.

To fully integrate spectral features, vegetation index features, and environmental features, this paper designs a Grouped Interaction Fusion Module (GIFM). Firstly, GateConv is employed to extract local and nonlinear representations for different features, followed by the AttBlock module designed based on the self-attention mechanism to extract global dependencies, thereby obtaining various features with high expressive power. Secondly, channel attention pooling is used to compress the high-dimensional features corresponding to the three categories into tokens with a dimension of 1, respectively, to reduce redundant information. Then, the three tokens are mapped back to each feature through addition, respectively, to supplement the internal representations of different features. Finally, the gated fusion method is used to adaptively assign weights to different features, achieving the efficient integration of multiple types of information. The detailed workflow is illustrated in Figure 4.

### 3.4. Apple Flower and Fruit Detection Model

Taking into account the representativeness of the YOLO series models, the balance between detection accuracy and inference efficiency, the differences in network structure and performance, and their applicability in complex natural scenes, this paper chose YOLOv11n as the benchmark model. This model has good computational efficiency while ensuring high detection performance. Its structural design is representative and has been widely applied in recent related research, which can serve as a reasonable benchmark for subsequent model comparison and improvement.

**Figure 5:**
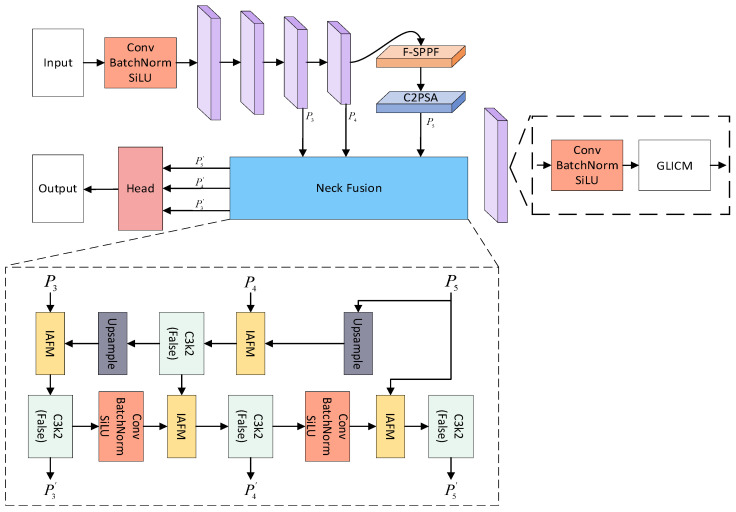
The framework diagram of YOLO-A model.

To improve the detection performance for apple flowers and fruits under complex orchard conditions, this study proposes a YOLOv11n-based detection model, referred to as YOLO-A. In the Backbone part, a Global–Local Information Capture Module (GLICM) and a Frequency Domain-Enhanced SPPF (F-SPPF) are designed. In the Neck part, an Interactive Attention Fusion Module (IAFM) is designed to replace the simple Concat operation and achieve feature fusion. The model framework is shown in Figure 5.

#### 3.4.1. Global-Local Information Capture Module

2D Selective Scan (SS2D) is an efficient two-dimensional state space scanning method proposed in the VMamba [25] model to address the quadratic complexity issue of the self-attention mechanism in Transformer. By extending the one-dimensional selective scan to a two-dimensional four-direction scan and integrating contextual information through state space model recursion, it achieves reduced complexity while capturing long-range dependencies.

**Figure 6:**
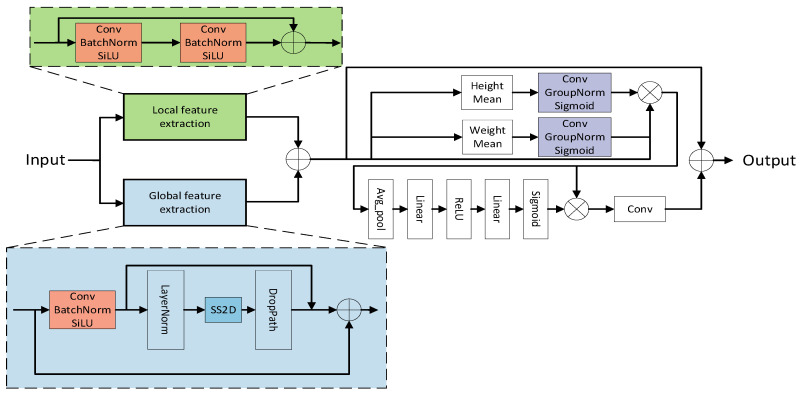
The structure diagram of Global–Local Information Capture Module.

To address the issue of excessively high computational complexity in extracting global features using the Transformer structure, this paper designs a Global–Local Information Capture Module (GLICM) by combining structures such as SS2D in VMamba and convolution. Firstly, the input features undergo a local feature extraction branch dominated by convolution operations, while also passing through a global feature extraction branch dominated by the SS2D module. Secondly, after extracting local and global features, the two are added together. Then, the summed result is fed into an axial-channel attention to enhance feature representation, thereby obtaining an enhanced feature representation that combines spatial direction sensitivity and channel selectivity. Compared to modeling methods that rely solely on Transformers or convolutions, GLICM can more efficiently balance global perception capabilities and computational efficiency in complex scenes. The architecture of the proposed GLICM model is presented in Figure 6.

#### 3.4.2. Frequency Domain-Enhanced SPPF

**Figure 7:**
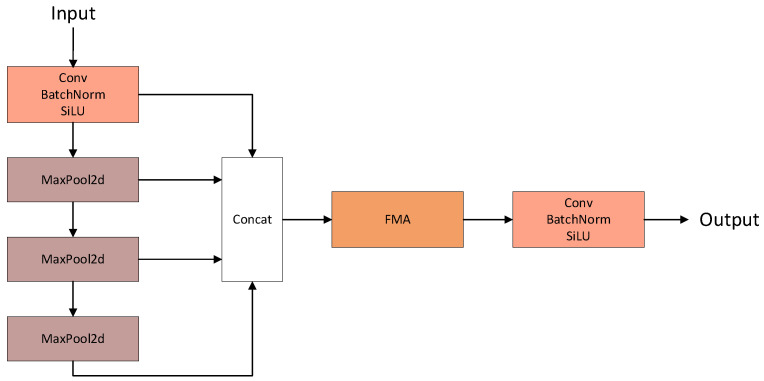
The structural diagram of Frequency Domain-Enhanced SPPF.

To efficiently extract features of different scales while maintaining efficiency, this paper designs a Frequency Domain-Enhanced SPPF (F-SPPF). Features with different receptive fields are obtained through convolution and continuous max pooling operations, concatenated, and further modeled for global dependencies using Fourier Modulated Attention (FMA) [26]. Finally, enhanced feature representations that combine multi-scale spatial information and cross-domain global dependencies are output through convolution and other operations. By introducing FMA, F-SPPF not only retains the efficient multi-scale feature extraction capability of traditional SPPF modules, but also enhances the perception ability of global structural information. The specific structure is shown in Figure 7.

#### 3.4.3. Interactive Attention Fusion Module

**Figure 8:**
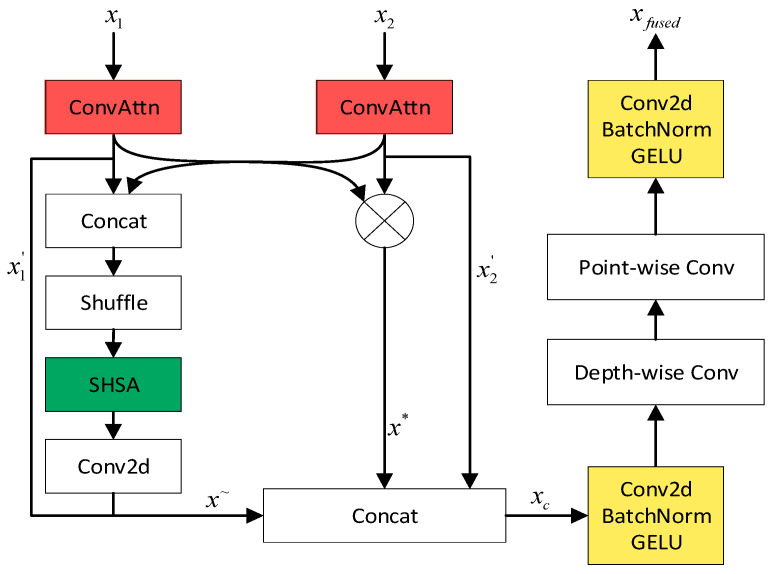
The structural diagram of Interactive Attention Fusion Module.

This paper proposes an Interactive Attention Fusion Module (IAFM) to address the problem of difficulty in characterizing the complementary relationship between features at different scales during the commonly used Concat operation for channel dimension stacking in multi-scale feature fusion. Firstly, the input features undergo internal enhancement through the Convolutional Attention Module (ConvAttn) [27], resulting in $x_{1}^{\prime }$ and $x_{2}^{\prime }$, as shown in Equation (1). Secondly, the enhanced features are concatenated and the restriction of fixed channel grouping is removed through channel shuffling. Then, a Single-Head Self-Attention mechanism (SHSA) is employed to capture the long-range dependencies of the features, further enhancing the expressive power of the features, resulting in $x^{\sim }$, as shown in Equation (2). Simultaneously, the enhanced features are multiplied to achieve explicit interaction between features, yielding $x^{*}$, as shown in Equation (3). Finally, $x_{1}^{\prime }$, $x_{2}^{\prime }$, $x^{\sim }$ and $x^{*}$ are concatenated, and through operations such as depthwise convolution and pointwise convolution, the final fused feature $x_{fused}$ is obtained. Compared with simple concatenation, this module can more effectively model the complementarity between multi-scale features. Figure 8 illustrates the detailed architecture of the proposed module.

(1) $$x_{1}^{\prime }=ConvAttn(x_{1}),\;x_{2}^{\prime }=ConvAttn(x_{2})$$

(2) $$x^{\sim }=conv(SHSA(Shuffle(concat(x_{1}^{\prime },x_{2}^{\prime }))))$$

(3) $$x^{*}=x_{1}^{\prime }\times x_{2}^{\prime }$$

(4) $$x_{c}=concat(x_{1}^{\prime },x_{2}^{\prime },x^{\sim },x^{*})$$

(5) $$x_{fused}=conv(BN(GELU(PWConv(DWConv(conv(BN(GELU(x_{c}))))))))$$

### 3.5. Yield Prediction Correction for Small-Scale Orchards

To obtain more accurate small-scale orchard yield prediction results, this paper delineates 30 fruit trees within the planting area of the Tianshui Fruit Research Institute as the local sampling area. The YOLO-A model is used to identify fruits individually for each fruit tree in the local sampling area. By combining the empirical single fruit weight, the actual true yield $Y_{true\_sample}$ corresponding to the local sampling area is obtained. The trained large-scale yield prediction model for districts and counties is applied to the local sampling area to obtain the predicted yield, denoted as $Y_{pre\_sample}$. The correction factor α can be derived from the two, as shown in Equation (6). Since the distribution of fruit trees in the sampling area is relatively uniform and consistent with the overall planting density of the orchard, the idea of proportional correction is adopted. After obtaining the predicted yield $Y_{pre}$ of the entire orchard through the large-scale yield forecasting model at the district and county levels, the corrected predicted yield $Y_{pre\_end}$ is obtained by combining the correction factor, as shown in Equation (7).

(6) $$\alpha =\frac{Y_{true\_sample}}{Y_{pre\_sample}}$$

(7) $$Y_{pre\_end}={\alpha \times Y}_{pre}$$

### 3.6. Flowchart and Pseudo-Code

**Figure 9:**
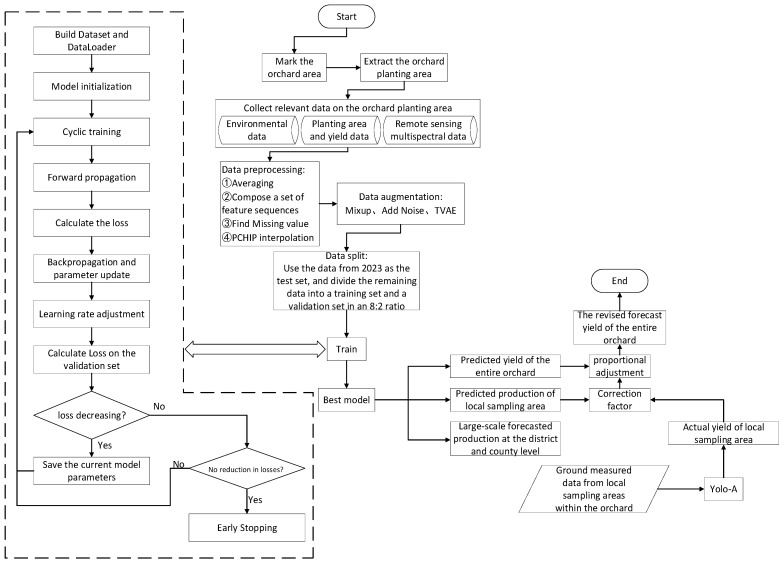
Flowchart.

The pseudo-code of the proposed apple orchard yield prediction framework is provided in Table 3, while its workflow is illustrated in Figure 9.

## 4. Results

### 4.1. Experimental Environment

The experimental environment configuration for this paper is shown in Table 4. The experimental hyperparameter information is shown in Table 5.

### 4.2. Experimental Results of Apple Orchard Planting Area Extraction

This paper uses arable land areas from land cover type data as the base map to distinguish between orchard areas and non-orchard areas. Firstly, to investigate how different feature combinations influence the model performance, this paper designs three feature combination methods, selects the random forest model as the basic model for classification, and chooses the corresponding results of Tianshui City for display, as shown in Table 6.

Table 6 indicates that the classification results obtained using Scheme Three are the most reliable. To further select a model with good performance, this paper employs Plan 3 (Elevation data + Sentinel-2 data and VI + Texture features) and combines it with five methods: Random Forest, SVM, Cart, GradientTreeBoost, and KNN for classification. The model performance is evaluated using OA, Kappa coefficient, and planting area difference. The experimental outcomes are summarized in Table 7.

It is evident from Table 7 that the Random Forest model achieves superior classification results. The results obtained by using the trained Random Forest model to extract orchard planting areas within Tianshui City from 2019 to 2023 are presented in Table 8.

As can be seen from Table 8, after screening different feature combinations and comparing models, the final results of fruit tree planting areas obtained in this paper are relatively reliable, and subsequent research can be conducted based on these results.

### 4.3. Large-Scale Yield Prediction Experiment at District and County Levels

#### 4.3.1. Experimental Results of Large-Scale Yield Prediction at the District and County Levels

For the performance evaluation of the proposed APYieldNet model, seven methods were selected for comparison: Lasso regression [15], Ridge regression [15], Multilayer Linear Regression [28], Partial Least Squares Regression (PLSR) [15], Long Short-Term Memory (LSTM) [29], Gated Recurrent Unit (GRU) [29], and Bidirectional Long Short-Term Memory (BiLSTM) [29]. Table 9 reports the experimental performance of the proposed method. In order to evaluate the statistical significance of the model performance and the stability of the results, this paper conducted 1000 bootstrap resampling on the test set. In each iteration, samples of the same size as the original test set were extracted with replacement, and each evaluation index was recalculated. The results in the table are presented in the form of “mean ± 95% confidence interval half width”, where “±” represents the half width of the 95% confidence interval estimated by Bootstrap, which can intuitively reflect the uncertainty of model performance and the differences between different methods.

#### 4.3.2. Ablation Experiment

To test the modeling capabilities of the TCN Layer and ASB used in the large-scale yield prediction model for districts and counties, this paper designed an ablation experiment. By eliminating or retaining different modules, we analyzed their impact on prediction performance. A summary of the experimental findings is provided in Table 10.

### 4.4. The Results of Apple Flowers and Fruits Detection

#### 4.4.1. Apple Flower Detection Results

**Figure 10:**
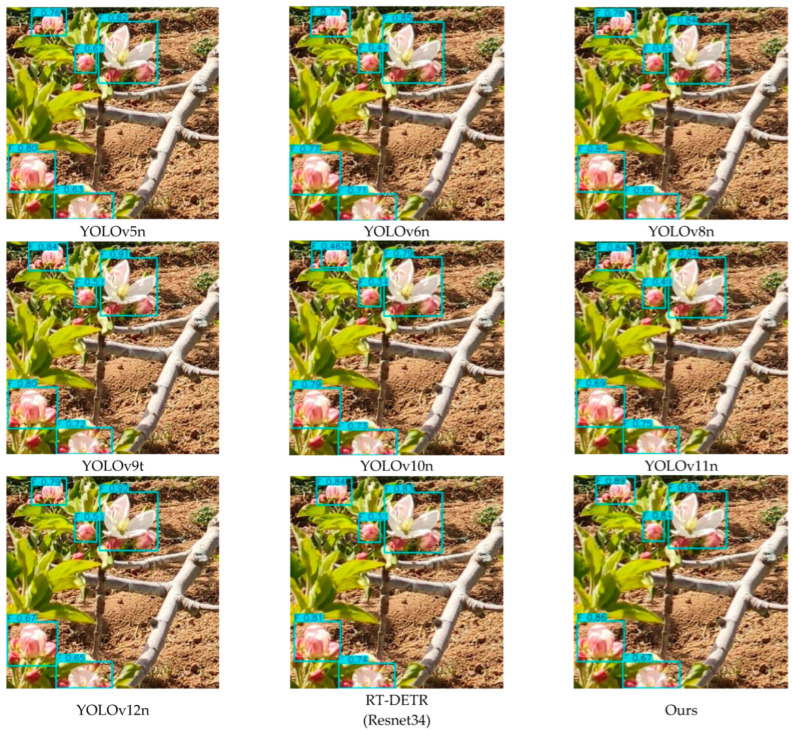
Apple flower detection results.

To investigate the detection capability of the proposed YOLO-A model for apple blossoms, we compared it with YOLOv5n, YOLOv6n, YOLOv8n, YOLOv9t, YOLOv10n, YOLOv11n, YOLOv12n, and RT-DETR (Resnet34). Table 11 reports the experimental performance of the proposed model. The detection results of different models for apple blossoms are shown in Figure 10.

#### 4.4.2. Apple Detection Results

**Figure 11:**
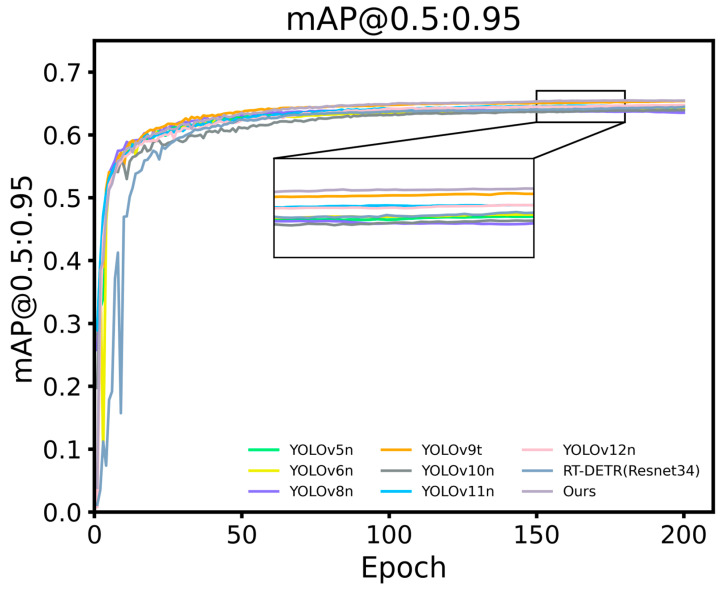
Comparison of model accuracy (mAP@50:95).

**Figure 12:**
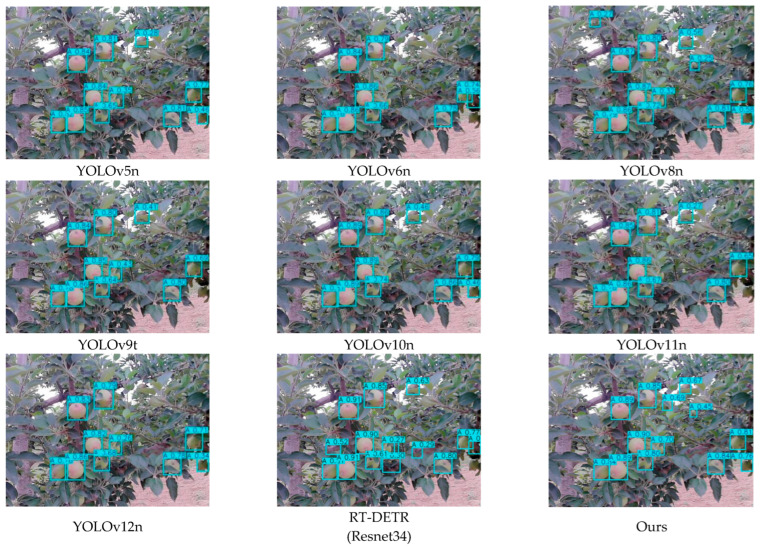
Detection results of apples in the young fruit stage.

**Figure 13:**
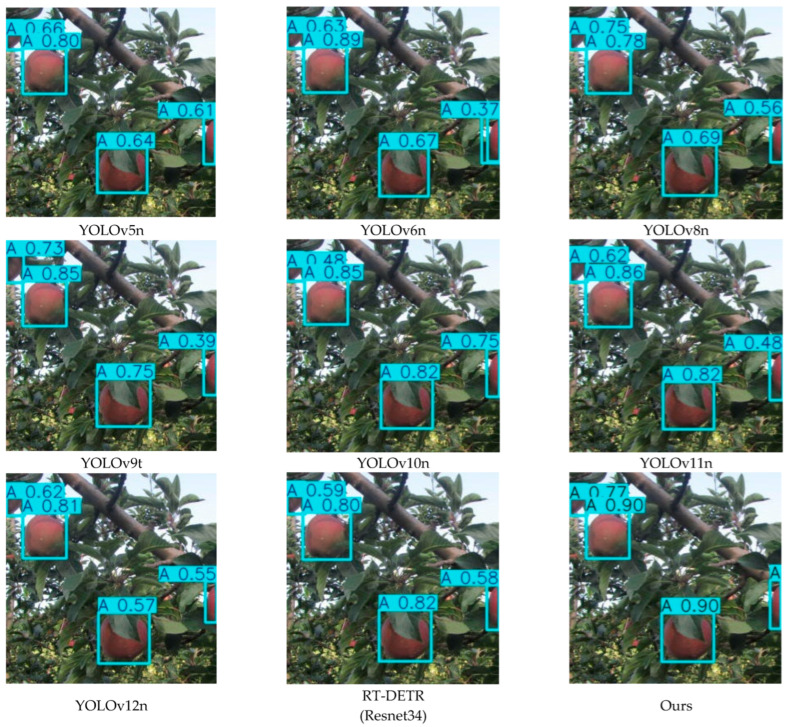
Detection results of apples during the fruit expansion stage.

**Figure 14:**
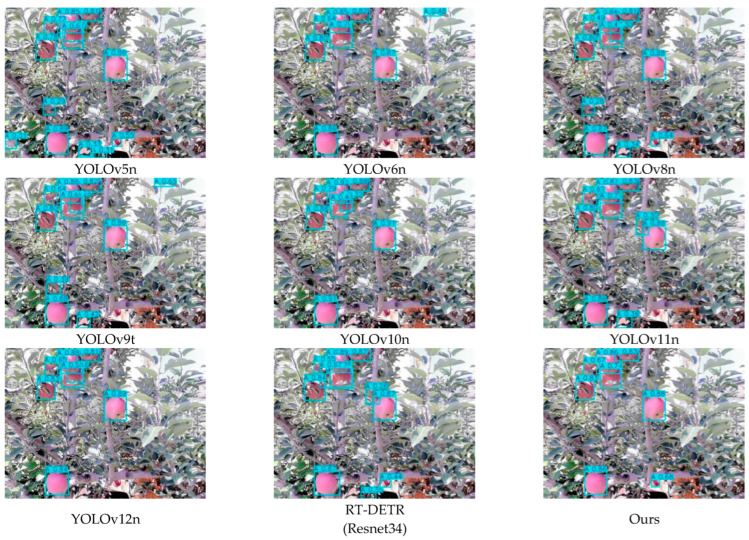
Detection results of mature apples.

To comprehensively evaluate the detection performance of the proposed YOLO-A model, we compared it with YOLOv5n, YOLOv6n, YOLOv8n, YOLOv9t, YOLOv10n, YOLOv11n, YOLOv12n, and RT-DETR (Resnet34). A summary of the experimental results is provided in Table 12. Figure 11 shows the performance of different detection models mAP@50:95 accuracy curve. The detection performances of different models at the young fruit, fruit expansion, and mature stages are depicted in Figure 12, Figure 13 and Figure 14, respectively.

**Figure 15:**
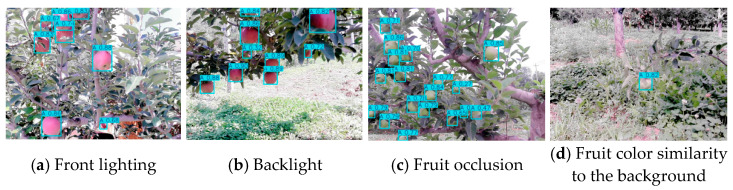
Apple fruit detection results in different complex orchard scenarios.

To further evaluate the detection performance of YOLO-A in real orchard environments, this paper visualized and analyzed the detection results of the model in typical complex scenes, such as different lighting conditions, fruit occlusion, and fruit color similarity to the background, as shown in Figure 15.

#### 4.4.3. Ablation Experiment of Apple Fruit Detection

To validate the performance improvement brought by the proposed YOLO-A model, this paper conducts ablation experiments on GLICM, F-SPPF, IAFM, and their different combinations. Table 13 reports the performance results from the ablation experiments.

#### 4.4.4. Efficiency Comparison of Detection Models

This paper conducts an efficiency analysis based on GFLOPs, parameter quantity, and model file size, with the results presented in Table 14.

To evaluate the operational efficiency of our model and benchmark model (YOLOv11n) during the training and inference phases, we separately calculated the training time for each epoch, as well as the time costs for pre-processing, model inference, and post-processing. The relevant results are shown in Table 15.

### 4.5. Correction Experiment of Yield Prediction in Small-Scale Orchards

In order to obtain more accurate small-scale orchard yield prediction results, this paper employs a proportional correction method to correct the predicted yields obtained from the yield prediction model by combining ground observation data. The ground observation data is divided into two categories: one is the estimation of yield per mu in the local sampling area based on the YOLO-A model, which involves apple fruit recognition, manual review, and empirical single fruit weight for 30 trees within the local sampling area of the Tianshui Fruit Research Institute in 2023; the other is the yield per mu of apples in the complete planting area of the Tianshui Fruit Research Institute, obtained through harvesting and weighing with precision instruments in the same year (used to measure the correction effect). The yield prediction results for the local sampling area and the complete planting area of the Tianshui Fruit Research Institute using the trained large-scale yield prediction model for counties are shown in Table 16.

**Figure 16:**
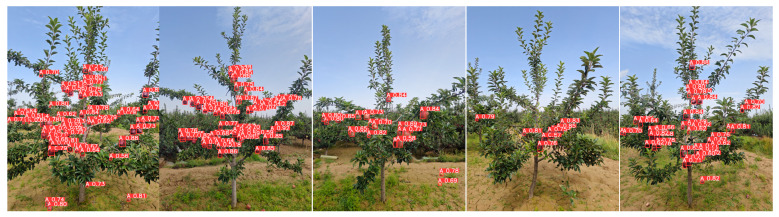
Display of fruit recognition results for some fruit trees within the sampling area.

Using the YOLO-A model presented in this paper, fruit recognition was conducted on 30 trees within the local sampling area, with some results shown in Figure 16. Through manual counting and verification combined with empirical single fruit weight, the actual yield per mu in the local sampling area was approximately 1500 kg/mu. Table 16 reports the revised yield prediction performance for small-scale orchards.

## 5. Discussion

### 5.1. Analysis of Large-Scale Yield Prediction Results at District and County Levels

#### 5.1.1. Analysis of the Results of Comparative Experiments on Large-Scale Yield Prediction at the District and County Levels

To demonstrate the regression prediction ability of the large-scale yield prediction model in this paper, a total of seven methods were selected for comparative experiments. The experimental results indicate that both our model and other models have a certain predictive ability. Traditional linear regression models include Lasso, multiple linear regression and PLSR. The model structure is relatively simple, but the MAE, RMSE, and MAPE are high, resulting in significant prediction errors. Among them, the Lasso model has the worst performance, with an MAE of 235.20 kg/mu, RMSE of 303.27 kg/mu, MAPE of 20.25%, and R^2^ of 0.68. The Ridge model is slightly better than the Lasso model, and the multiple linear regression and PLSR models further improved predictive performance, but there are still high errors. In deep learning models, the MAE, RMSE, and R^2^ of LSTM are 199.78 kg/mu, 270.82 kg/mu, and 0.73, respectively; GRU is further improved on the basis of LSTM, and although it can capture complex patterns in time series features during the growth period of apples, it still has a high prediction error. BiLSTM has bidirectional learning characteristics and can extract features from both forward and backward information of a sequence simultaneously. It has strong predictive ability, with an MAE, RMSE, and R^2^ of 187.52 kg/mu, 203.92 kg/mu, and 0.80, respectively. However, our model achieved better results than other models, including BiLSTM, with an MAE, RMSE, and R^2^ of 152.68 kg/mu, 203.92 kg/mu, and 0.85, respectively. Combined with Bootstrap resampling results, it can be seen that our model has strong regression prediction ability and is statistically superior to traditional linear models. At the same time, it also shows a certain performance improvement trend compared to deep learning baselines.

#### 5.1.2. Analysis of the Results of Ablation Experiments of Large-Scale Yield Prediction at the District and County Level

As can be seen from Table 10, whether introducing TCN Layer or ASB individually, the model performance significantly improves compared to the baseline: after introducing the TCN Layer, MAE decreased to 178.26 kg/mu, RMSE was 235.82 kg/mu, and R^2^ reached 0.81; while after introducing ASB, MAE and RMSE further decreased to 163.62 kg/mu and 222.22 kg/mu, respectively, and R^2^ increased to 0.83. When both play their roles simultaneously, the overall performance is the best, with MAE and RMSE decreasing to 152.68 kg/mu and 203.92 kg/mu, respectively, and R^2^ increasing to 0.85.

### 5.2. Analysis of Experimental Results for Apple Fruit Detection and Flower Detection

#### 5.2.1. Analysis of Apple Flower Detection Results

As summarized in Table 11, the YOLO series models achieve a better overall performance than RT-DETR (ResNet34) in apple flower detection, with YOLOv11n and YOLOv12n showing remarkable improvements in several evaluation metrics. Nevertheless, the proposed method delivers enhanced overall performance compared with existing approaches. In terms of Precision and Recall, the YOLO-A model in this paper achieves 79.17% and 76.74%, respectively. In terms of F1-Score, the result of YOLO-A is 77.94%, slightly higher than that of the superior YOLOv11n (77.78%), indicating that YOLO-A has an advantage in balancing Precision and Recall. In addition, the YOLO-A model in this paper achieves 83.13%, 56.86%, and 52.26% in mAP50, mAP75, and mAP50–95, respectively, all superior to other models.

#### 5.2.2. Analysis of Apple Fruit Detection Results

A comparative experiment involving multiple YOLO versions and the RT-DETR model was conducted to assess the apple recognition capability of the proposed YOLO-A model in complex orchard environments. As presented in the experimental results, the proposed YOLO-A model achieves higher Precision, Recall, and F1-Score than all compared models except RT-DETR, indicating enhanced detection stability. In terms of the mAP metric, the mAP50, mAP75, and mAP50–95 of the model proposed in this paper are superior to those of the other comparative models, with improvements of 0.98%, 2.84%, and 2.11%, respectively, compared to the original YOLOv11n model. This indicates that the model proposed in this paper has strong generalization capabilities under different IoU thresholds and can adapt to complex orchard detection scenes. Although the proposed model is slightly inferior to RT-DETR in Precision, its extremely high computational cost and large parameter count limit its application in practical apple orchard fruit detection scenarios.

The results indicate that YOLO-A can maintain a stable and accurate detection performance in most complex orchard scenes, demonstrating good robustness. The detection error is mainly concentrated in a few extreme cases, such as when the fruit is severely obstructed by leaves or branches, or in scenes where strong light reflection and deep shadows coexist.

#### 5.2.3. Analysis of Ablation Experiment Results for Apple Fruit Detection

Table 13 illustrates the effect of varying module configurations on the performance of the YOLO-A model in apple fruit detection tasks. According to Table 13, introducing any single module results in measurable improvements in model performance. When both modules are introduced simultaneously, the overall performance is improved compared to that of a single module. The model reaches optimal results in all indicators when GLICM, F-SPPF, and IAFM are combined. These findings indicate that multi-module integration strengthens feature representation and substantially improves detection performance.

#### 5.2.4. Analysis of Detection Model Efficiency Results

Table 14 shows that the YOLO series models exhibit low computational complexity and a small parameter scale. Evaluation results show that the YOLO-A model has a moderate computational cost, parameter scale, and model size, achieving overall performance comparable to YOLOv5n and YOLOv8n. Combined with its advantage in detection accuracy, YOLO-A balances computational and storage overhead while ensuring performance, demonstrating good practicality.

Compared with the benchmark model (YOLOv11n), this paper’s model introduces a more complex feature modeling mechanism while increasing certain computational overhead, resulting in a decrease in training and inference efficiency compared to the benchmark model. This indicates that the method proposed in this paper focuses more on enhancing feature expression ability in design, rather than prioritizing real-time performance as the main optimization objective. In offline processing-based application scenarios such as apple fruit quantity statistics and yield estimation, the improvement in detection accuracy and robustness by the model is often more critical than strict real-time constraints. Therefore, the method proposed in this paper has practical application value in such tasks.

### 5.3. Analysis of the Results of the Small-Scale Orchard Yield Prediction Correction Experiment

Table 16 reveals that the yield prediction model presented in this study exhibits underestimation in small-scale orchard yield predictions. Therefore, incorporating ground-based observation data to correct the prediction results is crucial for estimating yields in small-scale orchards. Through calculation, the correction factor is approximately 1.15, and the corrected yield prediction result is approximately 1548.90 kg/mu, which is closer to the actual yield value. This indicates that incorporating ground-based observation data can effectively mitigate the issue of underestimating high yields in small-scale orchard yield estimation using APYieldNet. However, the proportional correction factor is essentially a linear correction, and in scenarios where it is applied across years and regions, a single proportion factor may struggle to fully characterize complex yield variation relationships, and its stability still needs further verification.

### 5.4. Discussion Summary

(1) Summary of Comparative Experiment on Large-scale Yield Prediction at District and County Levels

By comparing seven different methods, it can be concluded that the large-scale yield prediction model for districts and counties constructed in this paper achieves higher-quality prediction results in regression prediction tasks. Evaluation results show that the proposed model surpasses other approaches in MAE, RMSE, MAPE, and R^2^, highlighting its strong fitting ability and stability in apple yield prediction.

(2) Summary of ablation experiment on large-scale yield prediction at district and county levels

To evaluate the contributions of the TCN Layer and ASB, this paper conducted ablation experiments with and without the TCN Layer and ASB. The results showed that both played a positive role and were complementary. When used in combination, the model exhibited notably stronger predictive regression capabilities.

(3) Summary of apple flower and fruit detection experiments

In complex orchard scenes, the proposed YOLO-A model demonstrates strong performance in apple flower detection, while also achieving effective fruit recognition at different growth stages. It achieves stable improvements in Precision, Recall, F1-Score, and mAP, verifying the model’s effectiveness and application potential.

(4) Summary of apple fruit detection ablation experiment

Ablation experiments were conducted to investigate the contribution of individual and combined modules to the improved YOLO-A model. The results showed that the improved model exhibited relatively stable improvements in metrics such as Precision, Recall, and F1-Score.

(5) Summary of efficiency analysis experiment for detection model

Compared with mainstream detection models, the proposed YOLO-A model achieves balanced computational efficiency, parameter scale, and model size. Simultaneously, the model achieved good results in detection performance, indicating that it achieves a reasonable balance between performance and overhead.

(6) Summary of the experiment on yield prediction correction in small-scale orchards

By using ground observation data to correct the predicted yield, the model achieves a closer match to actual yields, which improves the reliability of the predictions. These results emphasize the contribution of ground observation data to enhancing the accuracy of yield predictions in small-scale orchards. However, the applicability of this proportional correction method across years and different environmental conditions still needs further evaluation.

## 6. Conclusions

This paper addresses the issues of traditional apple yield prediction methods, which require extensive manual counting, are difficult to apply to large-scale orchards, and do not fully utilize multi-source remote sensing features. It proposes an apple orchard yield prediction framework that combines multi-spectral remote sensing features with ground features. The framework comprises two core components: large-scale yield prediction at the county level and small-scale orchard yield prediction correction. Regarding the large-scale yield prediction at the county level, this paper designs the APYieldNet model. Experimental evaluation indicates that the model captures temporal features throughout the apple growth cycle. This capability leads to accurate yield predictions. The trained APYieldNet model is applied to small-scale orchards to predict local sampling areas and the entire orchard planting area. After generating the prediction results, the proposed YOLO-A model is applied to count fruits in each local sampling area. Combined with empirical single fruit weight, the actual yield of the local sampling areas is obtained. The prediction results of the entire orchard planting area obtained by APYieldNet are corrected through a proportional correction method. Experiments show that introducing ground features can effectively address the underestimation issue in small-scale orchard yield prediction using APYieldNet. Despite its performance advantages, the YOLO-A model proposed in this paper has high computational overhead during the training and inference stages. In the future, techniques such as pruning and distillation can be attempted to further improve the real-time performance of the model. Meanwhile, the proportional correction method used in this paper is mainly focused on small-scale orchards in the experimental area. Future research can be conducted in different ecological environments and planting modes over multiple years to further verify its universality.

## Figures and Tables

**Table 1:** The remote sensing data and environmental data.

	Feature Name	Introduction
Sentinel-2 data	B2	Blue
	B3	Green
	B4	Red
	B5	Band within the red light range
	B6	
	B7	
	B8	Near-infrared band
	B8A	
	B11	Shortwave infrared band
	B12	
Environmental data	Evap_tavg	Evapotranspiration
	LWdown_f_tavg	Downward longwave radiation flux
	Psurf_f_tavg	Surface pressure
	Rainf_f_tavg	Total precipitation rate
	SoilMoi00_10 cm_tavg	Soil moisture (0–10 cm underground)
	SoilMoi10_40 cm_tavg	Soil moisture (10–40 cm underground)
	SoilTemp00_10 cm_tavg	Soil temperature (0–10 cm underground)
	SoilTemp10_40 cm_tavg	Soil temperature (10–40 cm underground)
	SWdown_f_tavg	Surface downward shortwave radiation
	Tair_f_tavg	Near-surface air temperature
	Wind_f_tavg	Near-surface wind speed

**Table 2:** The core parameters of the camera.

Parameter Name	Value
Total pixels	Approximately 14.5 million pixels
Effective pixels	Approximately 14.1 million pixels
Sensor	1/2.3-inch CCD
Optical zoom	14 times
Focal Range	5.0–70.0 mm (equivalent 35 mm film camera 28–392 mm)
Display screen	3.0-inch LCD screen with a resolution of 230,000 pixels
Image processor	DIGIC 4
Maximum photo resolution	4320 × 3240
Anti-shake function	Support optical image stabilization (IS system)
Video shooting	Supports resolutions such as 1280 × 720 pixels (approximately 30 frames per second)

**Table 3:** Pseudo-code.

A Prediction Framework of Apple Orchard Yield with Multispectral Remote Sensing and Ground Features	
Input	During the growth period of apples, there are various characteristics, including those at the large-scale level of districts and counties (x,Y), characteristics of the entire orchard ($x_{orchard}$), and characteristics of local sampling areas within the orchard ($x_{plot}$).
	Image of fruits at maturity in a partial sampling area of the orchard.
Output	Forecasted production at the district and county level on a large scale.
	Revised forecast yield of small-scale orchards.
1	Obtain the apple orchard planting area within the study region.
2	Collect corresponding spectral features, vegetation index features, environmental features, and statistical features to construct a feature set x. Collect actual yield labels Y to form training data $D_{train}\leftarrow x∥Y$. Take the data corresponding to 2023 as the test set $D_{test}\leftarrow x_{test}∥Y_{test}$.
3	$D_{train\_mixup}\leftarrow Mixup(D_{train})$; $D_{train\_noise}\leftarrow Add\;Noise(D_{train})$; $D_{end}\leftarrow TVAE(D_{train\_mixup}\cup D_{train\_noise})$.
4	$D_{train},D_{val}\leftarrow D_{end}$, $D_{train}⊃x_{train},Y_{train}$, $D_{val}⊃x_{val},Y_{val}$.
5	Initialize model parameters M.
6	for epoch = 1 to N:
7	${\hat{Y}}_{train}\leftarrow APYieldNet(x_{train};M)$.
8	$L\leftarrow MSELoss({\hat{Y}}_{train},Y_{train})$.
9	Backpropagation and update parameters.
10	Adjust the learning rate(ReduceLROnPlateau).
11	${\hat{Y}}_{val}\leftarrow APYieldNet(x_{val};M)$.
12	$L_{val}\leftarrow MSELoss({\hat{Y}}_{val},Y_{val})$.
13	if $L_{val}$< best_loss:
14	Save the model parameter as $M^{*}$.
15	else if $L_{val}$ did not improve within the maximum patience round:
16	EarlyStopping.
17	end
18	Large-scale forecasted production at the district and county level: ${\hat{Y}}_{test}\leftarrow APYieldNet(x_{test};M^{*})$. The predicted yield of the entire orchard: ${\hat{Y}}_{orchard}\leftarrow APYieldNet(x_{orchard};M^{*})$. Predicted yield in local sampling area: ${\hat{Y}}_{plot}\leftarrow APYieldNet(x_{plot};M^{*})$.
19	The number of mature fruits in the local sampling area is counted using the YOLO-A model, and combined with the empirical single fruit weight, the actual yield $Y_{plot\_true}$ of the local sampling area is further obtained.
20	$\alpha =\frac{Y_{plot\_true}}{{\hat{Y}}_{plot}}$, ${\hat{Y}}_{orchard}^{*}=\alpha \times {\hat{Y}}_{orchard}$
21	Output the large-scale predicted yield ${\hat{Y}}_{test}$ for districts and counties, as well as the revised predicted yield ${\hat{Y}}_{orchard}^{*}$ for the entire orchard

**Table 4:** Environment configuration.

Environment	Configuration
CPU	Intel(R) Xeon(R) Gold 6148 CPU @ 2.40 GHz
GPU	NVIDIA Tesla V100 32 GB*2
Python	3.8.20
CUDA	11.8
cuDNN	8.7.0
Pytorch	2.1.1 + cu118
Operating System	CentOS 8.5

**Table 5:** Hyperparameter information.

Yield prediction tasks	**Hyperparameters**	**Value**	Object detection tasks	**Hyperparameters**	**Value**
	Max Epochs	500		Epoch	200
	Batch size	32		Batch size	24
	Optimizer	Adam		Image size	640
	Loss	MSE		Optimizer	SGD
	Learning Rate	1 × 10^−3^		Momentum	0.937
	LR Scheduler	ReduceLROnPlateau		Learning Rate	1 × 10^−2^
	Early Stopping patience	20		Weight decay	0.0005

**Table 6:** Evaluation of classification results using different feature combinations (2019).

	Elevation Data	Sentinel-2 Data and VI	Texture Features	OA	Kappa Coefficient	Difference in Planting Area (10,000 mu)
Plan 1	√	√	–	0.88	0.76	36.42
Plan 2	√	–	√	0.8	0.61	6.41
Plan 3	√	√	√	0.89	0.77	2.05

Note: The “–” symbol indicates that the data of that type was not used. The “√” symbol indicates that the data of that type was used.

**Table 7:** Evaluation of classification results using different methods combined with feature combination Plan 3 (2019).

	OA	Kappa Coefficient	Difference in Planting Area (10,000 mu)
Random Forest	0.89	0.77	2.05
SVM	0.65	0.3	33.23
Cart	0.83	0.65	93
GradientTreeBoost	0.86	0.71	9.91
KNN	0.8	0.59	30.19

**Table 8:** Orchard planting map extraction results obtained by the Random Forest model.

	2019	2020	2021	2022	2023
Actual area (10,000 mu)	99.86	101.21	103.95	108.23	110.15
Predicted area (10,000 mu)	101.91	103.58	102.69	105.3	114.25
Area difference	2.05	2.37	−1.26	−2.93	4.1

**Table 9:** Results of comparative experiments on large-scale yield prediction at the district and county level.

Model	MAE	RMSE	MAPE	R2
Lasso [15]	235.20 ± 56.71	303.27 ± 73.86	20.25 ± 6.35	0.68 ± 0.07
Ridge [15]	224.51 ± 54.29	288.32 ± 68.11	19.82 ± 6.53	0.70 ± 0.07
Multiple Linear Regression [28]	221.66 ± 48.53	278.54 ± 62.62	20.33 ± 6.89	0.72 ± 0.09
PLSR [15]	217.81 ± 47.85	276.91 ± 62.97	20.38 ± 7.45	0.72 ± 0.09
LSTM [29]	199.78 ± 58.38	270.82 ± 67.76	15.14 ± 4.36	0.73 ± 0.09
GRU [29]	196.00 ± 55.78	263.87 ± 72.94	15.48 ± 4.88	0.74 ± 0.11
BiLSTM [29]	187.52 ± 45.71	236.82 ± 54.61	14.78 ± 3.57	0.80 ± 0.07
Ours	152.68 ± 40.83	203.92 ± 57.28	12.64 ± 4.03	0.85 ± 0.06

**Table 10:** Performance outcomes from the ablation tests on the large-scale yield prediction model.

Module		MAE	RMSE	MAPE	R2
TCN Layer	ASB				
		177.79	240.29	15.34	0.79
√		178.26	235.82	13.51	0.81
	√	163.62	222.22	12.94	0.83
Ours		152.68	203.92	12.64	0.85

Note: The “√” symbol indicates that the use of this module.

**Table 11:** Detection metrics for different detection models.

	Precision	Recall	F1-Score	mAP50	mAP75	mAP50–95
YOLOv5n	77.59	73.65	75.57	81.62	55.64	51.52
YOLOv6n	78.5	74.8	76.6	81.88	55.81	51.27
YOLOv8n	78.5	74.47	76.44	81.94	56.3	51.52
YOLOv9t	75.34	77.01	76.16	82.35	57.01	52.23
YOLOv10n	75.52	76.2	75.86	81.66	55.96	51.03
YOLOv11n	78.24	77.33	77.78	82.71	56.24	51.92
YOLOv12n	76.61	74.85	75.72	81.66	56.79	51.47
RT-DETR (Resnet34)	73.07	69.41	71.19	74.35	51.76	47.81
Ours	79.17	76.74	77.94	83.13	56.86	52.26

**Table 12:** Evaluation results of detection metrics for various models.

	Precision	Recall	F1-Score	mAP50	mAP75	mAP50–95
YOLOv5n	90.79	88.97	89.87	95.02	72.06	62.33
YOLOv6n	90.85	87.79	89.29	94.61	72.06	61.85
YOLOv8n	90.26	90.02	90.14	95.26	72.9	62.74
YOLOv9t	92.05	88.73	90.36	95.32	74.02	63.08
YOLOv10n	91.45	88.76	90.08	95.13	74.13	63.54
YOLOv11n	92.17	88.52	90.31	95.3	73.69	63.21
YOLOv12n	90.68	89.75	90.21	95.47	74.05	63.36
RT-DETR (Resnet34)	93.84	89.24	91.48	95.68	73.14	62.83
Ours	93.03	90.22	91.6	96.28	76.53	65.32

**Table 13:** Results of apple fruit detection and ablation experiment.

Model			Precision	Recall	F1-Score	mAP50	mAP50–95
GLICM	F-SPPF	IAFM					
√			92.77	90.10	91.42	96.13	64.59
	√		92.73	90.27	91.48	96.20	64.74
		√	92.45	90.09	91.25	96.21	64.66
√	√		92.71	90.08	91.38	96.14	64.95
	√	√	92.65	90.37	91.49	96.22	64.69
√		√	92.71	89.81	91.24	96.11	64.71
Ours			93.03	90.22	91.6	96.28	65.32

Note: The “√” symbol indicates that the use of this module.

**Table 14:** Model efficiency analysis.

	GFLOPs	Para/M	Model File Size/M
YOLOv5n	7.73	2.65	5.0
YOLOv6n	13.01	4.49	8.3
YOLOv8n	8.75	3.15	6.0
YOLOv9t	8.23	2.09	4.4
YOLOv10n	6.70	2.29	5.5
YOLOv11n	6.48	2.62	5.2
YOLOv12n	6.49	2.59	5.2
RT-DETR (Resnet34)	87.4	29.99	57.9
Ours	7.8	3.94	8.0

**Table 15:** Comparison of time costs between our model and benchmark model in the training and inference Stages.

	Training Stage		Inference Stage			
	Time/Epoch (s)	Total Time (h)	Pre (ms)	Infer (ms)	Post (ms)	FPS (E2E)
YOLOv11n	42.09	2.34	0.54	4.12	0.77	184.32
Ours	192.05	10.66	0.53	9.59	1.29	87.46

Note: All models were trained under the same hardware, training configuration, and iteration count. FPS (E2E) includes pre-processing, model inference, and post-processing time.

**Table 16:** Data and results of the small-scale orchard yield prediction and correction experiment.

	Local Sampling Area		Correction Factor	Complete Planting Area		Revised Predicted Yield (kg/mu)
	Model-Predicted Yield (kg/mu)	Actual Yield Obtained From YOLO-A (kg/mu)		Model-Predicted Yield (kg/mu)	Actual Yield (kg/mu)	
Value	1302.08	≈1500	≈1.15	1346.87	≈1750	1548.90

## Data Availability

The datasets generated during and/or analyzed during the current study are available from the corresponding author on reasonable request.
